# Preface

**Published:** 1848-01-01

**Authors:** 


### PREFACE.

THE Editor begs leave to introduce to the notice of his readers the First Volume of the "JOURNAL OF PSYCHOLOGICAL MEDICINE AND MENTAL PATHOLOGY." In doing so, he tenders his thanks to those who have so kindly extended to him their literary assistance, in the furtherance of his effort to establish a periodical devoted to the discussion of questions in relation to the Human Mind in its abnormal condition.

It has been the undeviating object of the Editor to address himself more especially to the profession; and therefore he has carefully excluded from the pages of the Journal all articles which might be considered *ad captandum* in their character.

It is often extremely difficult to control the minds and pens of others; but the editor has been anxious, by a careful selection of topics for elucidation, and by a judicious supervision of papers written for publication, to admit nothing into the Journal which had not a practical bearing, and which did not inculcate some great truth in connexion with the important subject of Psychological Medicine.

The Editor must leave his readers to form their own judgment as to the success with which he has carried his design into execution.

The Editor feels highly flattered at the many kind letters of encouragement which he has had the honour of receiving, from some of the first and most distinguished members of the profession.

He *feels* that the attempt to excite a deep interest in this the most elevated department of the science of medicine, is appreciated by those standing in the foremost ranks of the profession —men of the highest order of intellect. This encourages him to proceed in his anxious and responsible undertaking. His labour, as a brother editor observes, must be " a labour of love." He is willing that such should be the case. If his object be attained—namely, that of giving an *impetus* to that branch of the science of medicine which is termed *Psychological*—he will feel himself more than compensated for any humble exertions he may have made to effect so desirable a consummation.

Sussex House, Hammersmith,
October, 1848.

